# A Review on Risk Factors of Postpartum Depression in India and Its Management

**DOI:** 10.7759/cureus.29150

**Published:** 2022-09-14

**Authors:** Aditi Shelke, Swarupa Chakole

**Affiliations:** 1 Community Medicine, Jawaharlal Nehru Medical College, Datta Meghe Institute of Medical Sciences, Wardha, IND

**Keywords:** paternal postpartum depression, depression in india, postpartum depression, mental health, treatment, postpartum complications

## Abstract

Postpartum depression is the term used for depression that predominates in the postpartum period, which is increasingly seen in research and clinical practice up to 1 year after delivery. Other symptoms commonly seen in women with postpartum depression include mood swings or lability and excessive worry about the baby. In addition, postpartum depression is often associated with anxiety disorders or significant anxiety symptoms. Women with a history of psychiatric illness are prone to postpartum depression. Postpartum depression is a crucial psychological health ailment that confers a vast degree of disability in females and is often associated with significant emotional, behavioral, and cognitive dangers in children. It is a disorder that is often unrecognized and undertreated. Postpartum depression is a critical issue to be addressed because it interferes with a woman’s self-care and parenting. It also affects a child’s mental growth and development. For these reasons, evaluation of risk factors is required to consider every facet of postpartum depression in women. This article reviews the associated risk factors and management of postpartum depression in India. Traditional studies for risk factors in postpartum depression have typically categorized women according to a particular stage of pregnancy that follows them into postpartum depression. Pregnancy-associated risk factors are estimated during pregnancy and are looked up for their predictive association with postpartum depression defined by clinical diagnostic methods or self-report assessment. Treatment options include psychotherapy and antidepressant medication. The risk of postpartum depression in fathers also follows maternal postpartum depression. Paternal depressive disorder is associated with adverse effects on child development. Early intervention for postpartum depression and anxiety may decrease the severity and recurrence of symptoms as well as the negative effects on the baby's health and development.

## Introduction and background

Over the past few decades, the burden of mental diseases has increased [1]. It has led to rising cases of psychiatric problems in people of various age groups [2]. Postpartum depression is a problem that affects women after giving birth. Psychiatric postpartum depression affects mothers shortly after giving birth to a child. It has become a common psychiatric illness in the past decade. Various risk factors are responsible for postpartum depression. Many women experience emotional symptoms during postpartum four to six weeks after delivery. Symptoms experienced in postpartum depression are mood lability, sadness, dysphoria, emotional confusion, and tearfulness [3]. The best window for postpartum depression screening is from two weeks to six months following delivery [4]. Proper management and treatment of postpartum depression are necessary for safeguarding the mother’s mental health. Postpartum depression determination is difficult due to postpartum changes in sleeping habits, eating habits, and tiredness.

Past psychiatric history of the women should be inquired about to rule out high-risk cases of women for postpartum depression because a) the depression history of a patient determines the prevalence of postpartum depression; b) past psychiatric conditions like bipolar disorder, obsessive-compulsive disorder, eating disorder, and schizophrenia increase the chances of postpartum depression; and c) the first two months of pregnancy is the crucial period for diagnosis of postpartum depression as compared to later months [5].

Many stress-causing factors can affect the development of postpartum depression. An increased risk of postpartum depression exists in people with depression following a past pregnancy. Untreated maternal depression can harm a baby's growth, and the mother-child attachment, and increase the risk that later kids will have anxiety and depressive symptoms [6]. It can cause marital distress in a marriage, issues in mother-child interaction, behavioral problems in children, etc. It can affect the mental health of the family indirectly [7]. According to the diagnostic and statistical manual of mental disorders (DSM-IV), the symptoms of postpartum depression begin within four weeks of delivery; however, women are at risk of postpartum depression for up to one year after the delivery. The Edinburgh postnatal depression scale (EPDS) is a validated tool for postpartum depression screening. Its widespread use raised the rate of postpartum depression diagnosis, from 3.7% before screening to 10.7% after the screening, with 19.8% of women having an abnormal screening test result [8]. Knowing the factors influencing the risk of postpartum depression and anxiety disorders can support early detection. These factors can occur during or after pregnancy, providing medical professionals with the opportunity to reduce risk factors at various stages and thereby prevent the mother from developing depression and anxiety. Postpartum depression has an impact on the child’s behavior which affect the child’s health, growth, and development [9]. Children's neurodevelopment and ensuing mental health are impacted by postpartum depression [10].

## Review

 A systematic review of 47 studies in 18 countries reported a prevalence of postpartum depression of 18.6% [11]. The risk of recurrence of postpartum depression is high in subsequent pregnancies [12]. Even non-depressive postpartum women regularly experience many symptoms that often indicate non-depressive women's depression, including fatigue, appetite disorders, and sleep disorders. Exercise may show some sort of improvement in the symptoms [13].

Risk factors in India

Stress: Many prominent factors in India such as poor living conditions, family disputes, crises, financial issues, more children to take care of, and fewer work opportunities are the factors influencing postpartum depression. Stressful events in postpartum are a sick baby, c-section birth, concern about body image, baby care, and unsatisfying delivery experience [14]. Major risk factors, such as demographic, social, environmental, biological, hormonal, and obstetric factors are responsible for postpartum depression. Family studies on psychiatric illness show a two to three times increase in the risk of depression for family members with depression based on closeness. Women with several co-morbidities like hypertension, diabetes mellitus, etc., are at risk of postpartum depression [15]. Financial hardships, having a female child, marital strife, a lack of family support, a history of psychiatric disease, high parity, pregnancy problems, and a lack of maternal education in India were risk factors for postpartum depression. Similar risk variables have been reported in earlier research from low- and middle-income nations [16].

**Table 1:** Risk factors for postpartum depression reported by studies included in the systematic review, India, 2010–2015. Based on [16].

Variable	Total cases	Reported cases of postpartum depression
1) Individual factors		
High maternal age	28	4
Current medical illness	6	2
History of psychiatric illness	11	8
Family history of psychiatric illness	13	7
Recent stressful life event	11	6
2) Marital relationship status		
Marital conflict	14	10
Domestic violence	8	6
Financial difficulties	21	19
3) Pregnancy-related factors		
Unwanted pregnancy	14	4
History of obstetric complication	18	3
Complicated pregnancy	22	8
High parity	23	9
Cesarean section	15	5

Nutrition: Pregnancy and lactation deplete nutrients essential to the neurotransmission system [17]. The quality of diet, proper dietary intake, and nutritional status significantly affect postpartum depression. Appropriate nutritional status has a positive impact on the mental health of the mother. One of those nutritional components is vitamin D, which has been claimed to help individuals with depression. Vitamin D in foods has been theorized to function as a neuroactive hormone. Numerous investigations have revealed that vitamin D receptors are widely dispersed in the human brain and that a vitamin D deficit affects neurotransmitters known to be implicated in depressive symptoms [18]. Decreased n-3 PUFA levels during postpartum have a significant impact on postpartum depression. The deficiency of n-3 PUFA alters the metabolism of dopamine. It can be a potential cause of postpartum depression. A properly balanced diet is necessary for the stable mental health of the woman. Proper nutrition keeps the physical health of women under check, which in turn influences the mental health of the women. A metabolic disorder during pregnancy also increases the chance of postpartum depression [19]. Due to the transfer of minerals to the fetus and newborn during pregnancy and nursing, women's bodies may become deficient in certain minerals. The trace elements zinc and selenium have been linked to depression [20].

Hormones: It is seen that reproductive hormones fluctuate rapidly after childbirth. It might increase the risk of postpartum depression in women. Hormone deficiency theory hypothesizes that deficiencies in estradiol and progesterone in vulnerable women can quickly lead to postpartum blues and depression [21]. However, while all women in India experience this change in hormone levels after childbirth, few women develop postpartum depression. Various other conditions such as preterm birth (less than 34 weeks gestation) and giving birth to a child with a congenital disability can also cause depression [22].

Past psychiatric history: The current best predictor of postpartum depression is the assessment of mental illness during the pre-pregnancy and pregnancy periods. Only 5% of women who had never had depression before or during pregnancy went on to experience postpartum depression, as opposed to 65% of those who had. History of unintended abortions can also cause an increase in the risk of postpartum depression [23].

Thyroid function: Tests that measure various aspects of thyroid function are readily available and widely used in India. Precisely, it measures thyroid-stimulating hormone (TSH), thyroxine (T4), and triiodothyronine (T3). Also, thyroid peroxidase (TPO) and thyroxine-binding globulin (TBG) indicate thyroid functions. However, timing is important when investigating the role of thyroid hormone in predicting postpartum depression as thyroid function responds to constant changes in other hormones during pregnancy [24].

Multiparous women: Women with more children are at a higher risk of developing postpartum depression in subsequent pregnancies. It is due to a higher psychological burden on the mother. Primiparous women have a higher level of self-acceptance than multiparous women, which enhances the psychosocial well-being of the women [25].

Anemia: Anemia is a common issue in India during pregnancy occurring due to iron deficiency [26]. It has been shown to exacerbate symptoms such as fatigue, irritability, and lack of concentration, all of these may affect mood after giving birth to a new mom, menstruation, and how she interacts with the child. Low hemoglobin levels in the first week of life were highly risk attributed to the development of postpartum depression [27].

Age: The young age of women during pregnancy is one of the risk factors for postpartum depression [28]. Most multiparous women of old age also face postpartum depression due to increased stress. It can be due to increased pregnancy complications. The highest number of cases of depression post-pregnancy are seen in women less than nineteen years of age [29].

Screening of postpartum depression

Postpartum depression has several adverse effects on the health of mothers. So screening is crucial for diagnosis of the same. Early screening helps in early intervention by clinicians. Screening of postpartum depression within four to twelve weeks of delivery can help in a better prognosis. Repeated screening for six to twelve months can help in the diagnosis of late-onset postpartum depression. Screening of depression symptoms is done using the Edinburgh postnatal depression scale (EPDS). It is a simple, 10-item measure that mothers may complete right away and assess. The scale goes from 0 (no symptoms) to 30 (severe depression and anxiety). To identify depression, a cutoff level of 10 is frequently utilized. This measurement is precise and reliable [30]. Women having few symptoms of postpartum depression often go undiagnosed. Screening helps in diagnosing such cases. Screening of postpartum depression may improve baby-care settings. Screening requires a clinician and his/her professional judgment regarding the health of the patient. The doctor should evaluate the depressed mother's level of depression and any modifications to her parenting style [31]. Home visits by nurses in India are done for screening of postpartum depression. Identification of the at-risk population is necessary for primary postpartum depression prevention, and early recognition of postpartum depression is necessary for secondary postpartum depression prevention. Screening for postpartum depression is effective when carried out by professionals in obstetrics/gynecology, pediatrics, or by public health nurses [32].

Management of postpartum depression

Appropriate treatment option for postpartum depression varies by how severe the woman's symptoms are, her functionality status, and her ability to relate to and care for the child. Symptoms ranging from mild to moderate can be treated in primary healthcare centers in India; however, a psychiatric referral is advised if the symptoms don't respond to primary management. Urgent referral is needed in severely ill cases, especially when self-harming thoughts or thoughts of harming others, psychosis, or mania are present. Unmanaged postpartum depression tends to have adverse and prolonged effects.

Healthcare professionals are crucial in the treatment of postpartum depression. They guide and counsel the pregnant female throughout the pregnancy and help in overcoming her fears after pregnancy. Numerous studies in India document a poor rate of prenatal depression screening, diagnosis, and treatment in the medical context. A doctor provides proper counseling to the patients to address their insecurities regarding the pregnancy. A visit to the clinician is an opportunity for the clinician to assess the clinical condition of the mother. Even women with mild symptoms of postpartum depression can recover with counseling [32,33].

**Figure 1:**
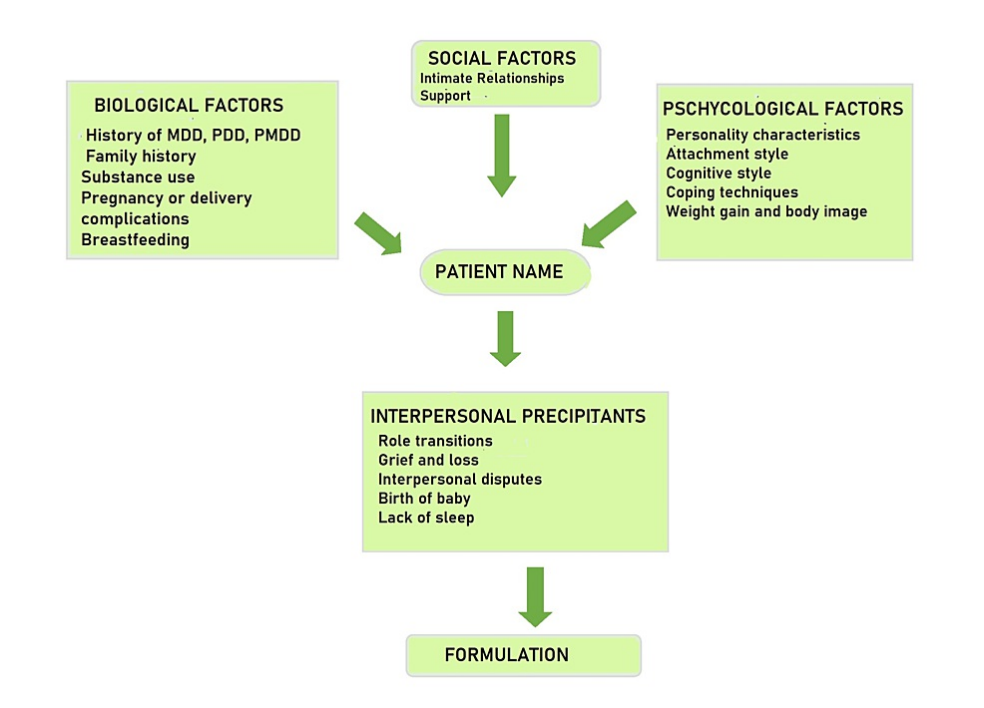
Formulation for postpartum depression. MDD, major depressive disorder; PDD, postpartum depression; PMDD, premenstrual dysphoric disorder. Based on [34].

Psychotherapy: A systematic review of postpartum depression treatment suggests that individual interpersonal therapy, cognitive behavioral therapy (CBT), and psychodynamic therapy can be effective psychotherapies for postpartum depression in India [33]. Some women have persistent urges to hurt their children, but they are reluctant to tell their loved ones about them. The therapy concentrates on the crucial interpersonal changes and difficulties postpartum women encounter. It takes a variable number of sessions, highlighting interpersonal disputes, role changes, or sorrows. It is a highly effective and short-term therapy. The therapy is problem-based and focuses on interpersonal stressors [34]. Figure 1 depicts the flow for formulation for postpartum depression.

 

Antidepressant therapy: Pharmacological therapy (selective serotonin reuptake inhibitor [SSRI] is used as a first-line drug) is prescribed for severely depressed individuals in cases where they do not show adequate response to non-pharmacological therapies or according to patient preference. Paroxetine and sertraline are the most commonly used SSRIs. It is safe for breastfeeding also. For a case of new-onset postpartum depression, sertraline is chiefly prescribed as first-line treatment because it is excreted in breast milk in minimal amounts. The most successful method of treating postpartum depression was through the use of medications and telemedicine [35]. Women should continue treatment for at least 9 to 12 months after experiencing a remission of symptoms, according to general depression treatment recommendations. In complex or treatment-resistant situations, augmentation with benzodiazepines, antipsychotics, and mood stabilizers may be required. The only alternative pharmacological agents for postpartum depression are omega-3 polyunsaturated fatty acids (PUFAs). Docosahexaenoic acid (DHA) and eicosapentaenoic acid (EPA) are the preferred sources of omega-3 PUFAs as they are the most biologically available [36]. Intravenous brexanolone (BRX) is an allopregnanolone solution that regulates GABA A receptors, restores levels in the third trimester, and enables receptor adaptation and symptom improvement [37]. Some women with postpartum depression may be given additional benzodiazepines for anxiety and insomnia. Breastfeeding infants exposed to benzodiazepines have been reported to have sedative effects and poor feeding, and low doses are recommended [38].

Other risk factors for postpartum depression include having experienced sexual abuse, having a poor attitude regarding previous pregnancies, and going through significant life events [39]. Also, contributing factors to the emergence of postpartum depression include resistance to the baby's gender and women's diminished self-esteem. Dangerous pregnancies also affect the outcome of postpartum depression. Postpartum complications can also cause stress in the woman [28]. Mismatches between the mothers' expectations and pregnancy events are factors that influence the development of depression. Women who wish to deliver by normal vaginal delivery during the perinatal period but who give birth by cesarean section are more prone to the risk of postpartum depression than other women [40].

Postpartum Depression in Fathers

In the postnatal period, it has been shown that maternal depression is the strongest predictor of paternal depression. A history of severe depression and high prenatal symptom ratings for sadness and anxiety have been found as the main predictors of paternal depression in the postnatal period. A personal history of depression is also associated with depression in fathers following the birth of their children [41]. In addition, depressed men are more likely than depressed women to engage in avoidant or hyperactive behavior, interpersonal problems, and poorer impulse control. The beginning of maternal depression during pregnancy, marital discontent, perceived stress levels, and personal characteristics, particularly personality traits and childhood experiences, have all been connected to the emergence of paternal depressive symptoms [42]. There may be a link between the postpartum depression of fathers and the absence of father-infant contact. The first few months after giving birth are crucial for the formation of the father-child bond. It is crucial to comprehend the nature of challenges in father-infant relationships in the context of postnatal depression due to the potential consequences for the child's long-term development. Therefore, physicians need to be aware of both the potential hazards to the growing baby and the risk of depression in new fathers [43].

## Conclusions

Postpartum depression is the most common healthcare issue in India faced by women who recently underwent pregnancy. Because of the potential consequences of untreated depression in the woman and her family, the healthcare worker needs to strive for an initial diagnosis and management of postnatal depression. The risk factors for postpartum depression include a history of psychiatric illness, stress, marital conflicts, complications in pregnancy, and financial difficulties. Pregnant women having a history of depression must be assessed for advisory prevention care for postpartum depression recurrence. Untreated postnatal depression affects the woman directly and her family indirectly. Management of postpartum depression is based on the patient's medical history, symptom severity, effects on functions, patient preference, and availability of resources and expertise. Women ranging from mild to moderate symptoms of depression undergo psychotherapy, cognitive behavioral therapy, interpersonal therapy, psychodynamic psychotherapy, or non-directive counseling. Treatment of postpartum depression includes the use of antidepressant medications such as SSRIs. To avoid recurrence, mothers with a personal history of postpartum depression should get counseling and strict supervision. Paternal postpartum depression in fathers is associated with withdrawn father-infant relationships. This can indirectly affect the growth and development of children. The management of postpartum depression is an important thing to safeguard women and their children in India.
